# Making the most of the waiting room: Electronic patient engagement, a mixed methods study

**DOI:** 10.1177/2055207617751304

**Published:** 2018-01-10

**Authors:** Victoria Coathup, Teresa Finlay, Harriet JA Teare, Jane Kaye, Matthew South, Fiona E Watt, Raashid Luqmani

**Affiliations:** 1 Centre for Health, Law and Emerging Technologies, 6396 University of Oxford, UK; 2 Oxford Centre for Human Brain Activity (OHBA), Department of Psychiatry, Warneford Hospital, 6396 University of Oxford, Oxford, UK; 3 Arthritis Research UK Centre for Osteoarthritis Pathogenesis, 6396 University of Oxford, UK; 4 Nuffield Department of Orthopaedics, Rheumatology and Musculoskeletal Sciences, 6396 University of Oxford, UK

**Keywords:** Musculoskeletal, patient participation, patient engagement, digital technology, research registry, waiting room, electronic health records, patient experience, consent, mobile applications

## Abstract

**Objective:**

The purpose of this study was to explore whether patients with musculoskeletal conditions would agree to use digital technologies to learn about research registries and make a decision about signing up whilst in the clinic waiting room.

**Methods:**

Patients were recruited from four hospital clinics across Oxfordshire. We used an explanatory mixed methods design with two sequential phases comprising an exploratory, cross-sectional questionnaire (*n* = 84), followed by focus group interviews (*n* = 8) to provide context for the findings from the questionnaire. Multivariate ordinal logistic regression models were used to explore relationships between patient preferences and characteristics. Thematic analysis was used to understand the reasons for patient preferences regarding digital technologies and research registries.

**Results:**

As participants' age increased, they were more likely to report a preference for face-to-face recruitment methods compared to those using digital technologies. Findings from the focus groups indicated this was primarily due to a fear of technology and physical limitations associated with a patient's condition. Patients also reported a preference for making a decision about signing up at a later date, which was attributed to patients feeling distracted whilst in the waiting room due to anxieties related to their upcoming appointment.

**Conclusions:**

Many patients with musculoskeletal conditions in the UK may be interested in learning about opportunities to participate in research whilst using digital technologies within the waiting room. The results suggest the need for choice regarding the presentation and format of information and whether it can be accessed at a later date at home.

## Introduction

The collective burden to the National Health Service (NHS) of musculoskeletal conditions is difficult to estimate,^1^ but with an ageing population it is likely to increase. A report published by Arthritis Research UK focusing on the 13 most common musculoskeletal conditions, estimated a prevalence of approximately 22% in females and 16% in males in the UK.^2^ Research is crucial for advancing understanding and management of musculoskeletal conditions, and the potential value of studying long-term outcomes from this population is significant.^3–5^ For this reason a decision was made to establish a musculoskeletal patient registry at the Nuffield Orthopaedic Centre, Oxford. The Oxford Patient Research Registry – Musculoskeletal (OxPaRRM) could also be used to combine various forms of clinical data for research purposes.

Patients with long-term conditions, including musculoskeletal conditions, require regular follow-up in specialist outpatient departments, satellite clinics and surgeries for monitoring of their symptoms and possible adjustment of therapy. Waiting rooms are also often used as a recruitment site for research. Therefore, it was thought it would be beneficial to recruit patients in clinic waiting areas. Waiting times in outpatient departments and surgeries can be lengthy, ranging from 10 minutes to two hours.^6^ This is of concern because evidence suggests that the longer patients wait for their clinic appointment, the less satisfied they are likely to be with their visit.^7^ However, waiting time could represent an opportunity to convey health education and related information, thus usefully filling patients' time whilst waiting; a recent study exploring the views of general practitioners (GPs) in France supported this view.^8^

Routine clinical care, including follow-up monitoring, generates a considerable amount of data including documentation of signs and symptoms, blood test results, biopsy and imaging findings, interventions and drug treatments, as well as demographic information. These data provide important information about the individual patient as well as being of potential value in studying the occurrence and aetiology of disease, possible risk factors and their frequency. The digitisation of medical records facilitates the storage, linking and sharing of these data, which can play a key role in the growing trend towards integrating clinical care and medical research. One method of moving towards this more integrated approach is through the use of healthcare patient registries. Registry-based health research uses data from healthcare databases, some of which were originally created for clinical, rather than research, purposes.^9^ A major strength of this research approach is that the data are already available, which minimises the time, cost and burden on patients and healthcare professionals.

The OxPaRRM project seeks to enrol patients with musculoskeletal conditions on a registry from which their demographic, diagnostic, clinical and treatment data can be accessed for epidemiological research into rheumatological conditions. Recruitment to the registry has relied on registered nurses (RNs) discussing the registry and inviting patients to participate while they waited for their appointment. To avoid duplication, RNs also check clinic lists prior to appointments to ensure that only patients who have not previously consented to the registry are approached. Whilst very successful (97% of invited patients agreed to sign up), this is a costly and time-consuming approach to recruitment; this study was conceived in order to ascertain whether a digital interface could replace the human interaction that currently results in successful recruitment to the registry.

Thus, the main aim of this study was to explore whether musculoskeletal patients would be happy to engage with digital technologies in the waiting room to find out about research registries and to decide whether or not to participate. A secondary aim was to establish what features were regarded as important when using digital technologies in the waiting room. This paper presents our mixed methods study of patients waiting for their appointment in rheumatology outpatient clinic areas.

## Methods

### Study design, setting and participants

We used an explanatory mixed methods design with two sequential phases composed of an exploratory, cross-sectional questionnaire, followed by focus group interviews to provide context for the findings from the questionnaire.^10^ This approach was specifically adopted to enable detailed exploration of issues related to the questionnaire and people's reactions to using software on an electronic tablet that mimicked enrolment on a research registry. The focus group interview enabled us to develop an understanding of patients' views about issues raised by the questionnaire that would not have been possible with the questionnaire alone^11^ and then use those views in discussing their experiences of using the tablet. This group interview format maximised the opportunity for developing common understandings through sharing experiences and discussion of different perspectives.^12^ All participants were attending a follow up visit within a publicly funded NHS hospital. Participants were eligible for inclusion if they were 18 years or older, with a diagnosed musculoskeletal condition and were attending one of four musculoskeletal outpatient clinics within the Oxford University Hospitals NHS Foundation Trust over a period of one month in November 2015. Upon arrival at the clinic, patients check in with a receptionist and are then directed to a seating area within the waiting room while they wait to be called to have blood samples taken. Once they have had blood samples taken, they are directed to wait in the seating area again until they are called for their appointment by the consultant.

### Recruitment

Clinicians identified eligible patients to be invited into the study. Two researchers (TF and VC) would then introduce themselves to the patient, explain the study and invite them to take part. If the patient agreed, the researcher would provide them with the study materials and collect the completed questionnaire as the patient was leaving the clinic. One clinic had a waiting room that was dedicated to the musculoskeletal outpatient clinic only. Therefore, upon arrival, the receptionist provided patients with a copy of the participant information sheet and questionnaire as they checked in for their appointment. Patients handed completed questionnaires back to the receptionist as they left the clinic. Consent was implied by the submission of a completed questionnaire.

The final page of the questionnaire provided information about the focus groups and invited patients to provide their contact details if they were interested in taking part. Interested patients were contacted by a member of the research team (VC) and invited to participate in a focus group. An invitation letter, participant information sheet and consent form were sent to patients. Upon confirming their participation, patients were sent confirmation details of the time, location and directions for the focus group.

### Questionnaire development

A literature review was conducted to explore the use of electronic devices within clinic waiting rooms and how the acceptability and feasibility of these tools were assessed. Important themes to be explored were then developed through discussions with all members of the research team, including specialist clinicians, research scientists and software developers. Three key themes were identified for the survey which included: whether patients would engage with an electronic device in the waiting room; what type of electronic device would be preferable; and how long patients would like before making a decision about signing up to a research registry. A short survey was initially drafted and reviewed by all members of the research team. One question was deemed too complex and broken down into a number of shorter questions. Because the questionnaire would be completed while patients were in the waiting room, it was essential that it could be completed within 5–10 min; therefore, members of the research team were timed as they read through and completed the questionnaire. The questionnaire was reviewed again and minor changes were made to the language to ensure simplicity and clarity. The final version was reviewed and approved by all members of the research team and comprised seven closed and two open questions, and included Likert scale responses with six categories (Very happy/Happy/Neither happy nor unhappy/Unhappy/Very unhappy/I don't know).

### Prototype research registry enrolment tool

A prototype Web application was developed for delivery via tablet, in full screen mode, and used in the study. The application allowed access via a pre-defined list of clinic attendees who authenticated with the combination of their surname, date of birth and gender. Upon authentication, new users were taken through a series of screens that replicated the existing paper consent process. Existing users could review their consent history, update their current consent and review their contact details.

### Focus groups

Two focus groups were conducted in January 2016. The purpose of the focus groups was to explore people's experiences of the waiting room and their views about enrolling on research registries using digital technologies while waiting for a consultation. Each focus group was facilitated by one researcher (TF) whilst another noted people's positions in the room, non-verbal interactions and audio-recorded the discussion (VC). Two software developers joined the focus group to present a simulated research registry enrolment tool on electronic tablets for use in waiting rooms and to guide participants through trialling it. This is also a methodology that was used in a study we completed on biobanking.^13^

The first aspect of the discussion explored people's experiences of waiting for consultations, their views on gaining knowledge about medical research for potential participation and their use of digital technologies. The tablets with the prototype research registry software were then demonstrated by the web developers following which each participant tested the tablet and software by reading the application's contents and setting up a dummy account on the fictional research registry. The latter part of the discussion focused on the design of the software, use of the tablet, its feasibility for use to engage people in medical research registries and its use in a waiting room setting. Suggestions for improvement on each of these aspects were also sought.

### Data analysis

Likert scale categories were collapsed, from six (Very happy/Happy/Neither happy nor unhappy/Unhappy/Very unhappy/I don't know) to four categories (Happy/Neither happy nor unhappy/Unhappy/I don't know) due to low numbers of responses within the extreme categories. Participant responses were summarised as frequencies and percentages, and the distribution of data was explored using histograms and two-by-two tables. Chi-squared test for independence was used initially to explore bivariate associations between categorical questionnaire variables.

Ordinal logistic regression models were used to explore relationships between participants’ views on face-to-face and electronic recruitment methods in the waiting room and participant characteristics (age and gender). In addition to this, ordinal logistic regression models were used to explore participant preferences (type of digital technology used in the waiting room and the time taken to make a decision about signing up) and participant characteristics. The response category of ‘I don't know’ was excluded in regression analyses and Likert scale responses were treated as ordinal, dependent variables and participant characteristics were treated as categorical, independent variables. Data analysis was conducted using STATA 14.^14^

Audio recordings of the focus groups were transcribed by a commercial organisation and the transcripts then checked against the audio recordings and notes of the proceedings by one researcher (VC). Transcripts were read by TF, VC and HJAT. An inductive approach was taken to coding the data where identification of codes was driven by the content of the data (Braun and Clarke, 2006).^15^ Focus group data were initially coded by VC using NVivo10^16^ and TF separately. A coding framework was developed on the basis of reflexive discussion of both of these sets of codes, and the data re-coded according to the framework. Analysis by TF and VC using the coding framework resulted in five themes when grouped, including commonalities and contradictions; the thematic analysis was discussed with HJAT and finally refined.

Quantitative and qualitative data were integrated using the weaving approach, previously described by Fetters and colleagues.^17^ This approach involved comparing results from the survey and focus groups and then interpreting the findings on a theme-by-theme basis.

### Missing data

Participants were excluded from the current analysis if all responses to questions two (face-to-face sign up), three (electronic sign up) and four (digital technology preferences) were missing, because it was not possible to address the aims of this study. Otherwise, available data were entered and non-responses were coded as missing values for analysis.

## Results

Approximately 144 patients were invited to participate in the study while they waited for their clinic appointment. A total of 84 patients completed and returned a questionnaire, providing an approximate response rate of 58%. Of those who responded, 6% were already signed up to OxPaRRM and 14% were unsure. The majority of participants were female and over the age of 40 years (55%). Two focus groups were conducted with a total of eight participants, with each lasting approximately two hours. Both focus groups were conducted in a small meeting room within the hospital. Participant characteristics are presented in Table 1.

**Table 1.** Characteristics of sample population *n* (%).^a^

	Survey (*n* = 84*)^b^		Focus groups (*n* = 8)	
	Male	Female	Male	Female
Age				
≤40 years	2 (2)	13 (15)	0 (0)	0 (0)
41–60 years	10 (12)	27 (32)	1 (13)	2 (25)
≥61 years	11 (13)	19 (23)	2 (25)	3 (38)
Total	23 (27)	59 (70)	3 (38)	5 (62)

a Percentage of sample population within each study.

b Not all cells add up to 84 due to missing data.

During the integration phase of the analysis, three broad themes became apparent across the quantitative and qualitative data. The first theme, ‘The personal touch’, encompassed participants’ views on receiving an invitation face-to-face or via digital technology in the waiting room. Three sub-themes were also identified: ‘Putting names to faces’; ‘Identity as a musculoskeletal patient’; and ‘Research findings’. The second theme, ‘Technology preferences’, comprised participants’ views on using different types of digital technology in the waiting room and what influenced their preferences. Two sub-themes were also identified: ‘Fear of digital technology’; and ‘Physical limitations’. The final theme, ‘Place and time’, covered issues pertaining to when and where participants’ would prefer to make a decision about signing up to a research registry, including one sub-theme, ‘Distractions in the waiting room’.

### The personal touch

The first theme that became apparent in the data was the choice between face-to-face or electronic invitations to join a research registry, and the factors that influence patients' decisions. A slightly higher proportion of participants reported that they would be interested in signing up to OxPaRRM if they were invited face-to-face, compared to an electronic invitation in the waiting room (Table 2).

**Table 2.** Participant interest in the Oxford Patient Research Registry – Musculoskeletal (OxPaRRM) by type of invitation (*n* = 83).^a^

	Total (*n* = 83)^a^	Females (*n* = 56) Age (years)			Males (*n* = 22) Age (years)		
		≤40	41–60	≥61	≤40	41–60	≥61
	*n* (%)	*n* (%)	*n* (%)	*n* (%)	*n* (%)	*n* (%)	*n* (%)
Interested in face-to-face invitation							
Yes	32 (38)	0 (0)	11 (20)	10 (18)	1 (5)	4 (18)	4 (18)
Maybe	43 (52)	11 (20)	13 (23)	8 (14)	1 (5)	5 (23)	2 (9)
No	8 (10)	2 (4)	0 (0)	1 (2)	0 (0)	0 (0)	5 (19)
Interested in electronic invitation							
Yes	30 (36)	1 (2)	12 (21)	6 (11)	2 (9)	3 (11)	5 (23)
Maybe	41 (49)	12 (21)	11 (20)	7 (13)	0 (0)	5 (19)	2 (9)
No	12 (15)	0 (0)	2 (4)	5 (9)	0 (0)	1 (5)	4 (18)

a Not all cells add up to 83 due to missing data.

Participant responses were also investigated in relation to age and gender. Approximately 80% of participants who were ≤40 years old reported being unsure about whether they would sign up to OxPaRRM or not, regardless of the medium of invitation. Ordinal logistic regression models also indicated that being interested in signing up if invited face-to-face by a nurse was significantly associated with older age. Participants aged 41–60 years were approximately five times more likely to be interested in signing up to OxPaRRM if invited face-to-face, compared to those who were ≤40 years old (odds ratio (OR) = 5.28, 95% confidence interval (CI) = 1.52–18.31; *p* = 0.009) and participants aged 61 years or older were 3.5 times more likely to sign up to OxPaRRM if invited face-to-face by a nurse, compared to those who were ≤40 years old (OR = 3.56, 95% CI = 0.99–12.76; *p* = 0.05). There were no significant differences between age groups when participants were asked about their interest in OxPaRRM if invited using an electronic device in the waiting room. There appeared to be no relationship between a person's interest in OxPaRRM and gender.

### Putting names to faces

During the focus groups, participants' choices between a face-to-face and electronic invite were discussed and it became clear that participants valued the personal interaction and felt that a face-to-face invitation would be more likely to provide this. For some patients, this could be a deciding factor as to whether or not they signed up to the registry.

If you're approached by somebody personally, there's maybe more of a chance of signing up with a personal approach than somebody on a screen, tick box here. Maybe I wouldn't. I'd need some understanding and prompting. I think the personal touch would come across better, possibly.

Given the association of less interest in digital technology with older participants, this reticence towards signing up using a digital interface could be based on lack of experience of using one, or a perceived lack of confidence in doing so. Additionally it could be related to a perceived loss of the additional tailored information that questions and answers in a face-to-face recruitment scenario provide.

When discussing how electronic devices’ content could be designed to provide a more personal touch, it became clear that knowing who was running the study and why, were important factors for participants in the focus group:

‘The people who are actually doing it… Like names to faces, it is not just a… Do you know what I mean…It is a bit more personal. This is the Head Researcher or whatever they do’.

Two participants, independently in two separate focus groups, suggested the use of video clips as a way to provide the personal touch and get the necessary information across in a way that is engaging whilst in the waiting room.

Or a short video of someone, a You Tube clip of some sort, yes, some interaction or a doctor explaining whatever it was in a short clip would be interesting if it was sold in the right way that captured your imagination. Yes, it just needs to be sold in the right way, doesn't it?

So what we were saying about that being perhaps impersonal, it might be quite nice having the Head of Research maybe not just a picture but them, a little short video saying, ‘Thank you very much for considering consenting to be part of this research programme. The main aim of the research programme is…’. So you have got a visual impact for the person who is taking that in.

This suggests that personal involvement of researchers (or their associates including the RNs who currently recruit patients to the registry) is important in developing patients' interest in participating in the research registry.

### Identity as a patient with a musculoskeletal condition

Another key theme was the importance of connections and networks with other patients with musculoskeletal conditions, particularly for those with less common conditions, such as vasculitis. Participants identified strongly with others with similar diagnoses, as they both described and demonstrated during the course of the focus groups.

It is nice to meet up with other people because what we have got is stressful. It is coming to terms with a lifelong chronic issue and it is great to speak to people who are going through the same thing.

One participant suggested that information about OxPaRRM might provide a starting point to connect with other patients in the waiting room. Whilst the following excerpt raises confidentiality issues it nonetheless demonstrates the importance to some patients of having an opportunity to connect with others with similar problems.

I mean I am quite outgoing and speak to anyone but not a lot of people are, and perhaps just reading a leaflet and then it gives [a clue about the person]. ‘Oh look, oh they must have the same as me because they’ve picked up one of those leaflets there’.

These descriptions and behaviours seem indicative of the importance of social networks that develop around conditions due to a common experience of a disease. Other patients appeared to represent an important source of peer support and the waiting room was described by some as providing an opportunity to make connections with people experiencing similar problems.

### Research findings

While only three participants had taken part in a research project prior to this study, all participants contributed to the discussion on medical research. Participants were very supportive of medical research and acknowledged the importance of it for rheumatology. As this topic of discussion unfolded, it became clear that while participants were supportive of research, they would like more active engagement. They described expectations of more information about the progress and final outcomes of future studies they might consider participating in as well as detail about how their data would be used.

You know, it is like that thing with that [specific trial] research, go in there every month, ‘Oh thank you for your time’. But I still don't know what the outcome was. How good a job I did…, all those times I went up and they jabbed me in the arm with a needle and then I had flu for a week, did it come to a satisfactory conclusion?

And it is quite interesting to know, well at the end of the day what is going to happen to all this [information], how long are they going to hang on to all this information? What happens to it all at the end of the day?

The expectation of receiving feedback about study results is congruent with UK research funding bodies' policies to engage the public with research. They advocate this be achieved in part by making lay summaries of study publications available to participants and the public.^18^ However, there is the potential to use digital technologies to improve this feedback to patients.^19^

### Technology preference

**Figure 1.**
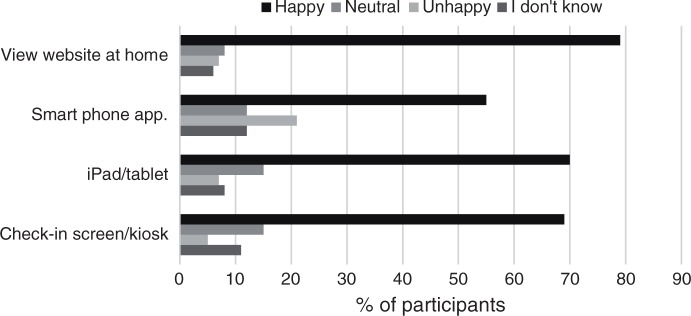
Participant views of using different types of digital technology to access the Oxford Patient Research Registry – Musculoskeletal (OxPaRRM) (*n* = 84).

Participants were asked about the type of digital technology they would prefer to use in the waiting room (Figure 1). Approximately 70% of participants reported being happy about using a check-in kiosk in the waiting room to access information about OxPaRRM, and similar views were reported in relation to the use of a tablet. Interestingly, views differed when asked about the use of a smartphone app in the waiting room; fewer participants were happy regarding this suggestion, and more reported being unhappy with the use of this device in the waiting room compared to any other digital technology. Overall, viewing a website at home was the most favoured option, with approximately 80% of participants reporting to be happy to access information about OxPaRRM at home via a website (Figure 1).

When participants' views were explored in relation to age and gender, it appeared that the type of digital technology used in the waiting room was associated with participant age. Results from the ordinal logistic regression models are presented in Table 3. As age increased, participants stated that they would be less happy to use a check-in kiosk in the waiting room to access information about OxPaRRM (OR = 0.13, 95% CI = 0.02–0.74; *p* = 0.022). A similar relationship was observed for tablets (OR = 0.08, 95% CI = 0.01–0.73; *p* = 0.025). No relationships were observed between the type of digital technology and gender. This suggests that this may change over time as younger generations more conversant with digital technologies constitute a larger proportion of the adult, older population suffering from a musculoskeletal condition. While this transition occurs, different kinds of strategies need to be used; face-to-face as well as digital options, and Web-based that can be used at home and else-where.

**Table 3.** Participants views on the use of different types of digital technologies in the waiting room by age and gender (*n* = 84).

	Check-in kiosk			Tablet			Smartphone			Website		
	OR	95% CI	*p*-Value	OR	95% CI	*p*-Value	OR	95% CI	*p*-Value	OR	95% CI	*p*-Value
Age (years)												
≤40		(ref)			(ref)			(ref)			(ref)	
41–60	0.45	(0.08–2.51)	0.365	0.22	(0.02–2.02)	0.181	0.76	(0.20–2.96)	0.694	0.40	(0.07–2.24)	0.297
≥61	0.13	(0.02–0.74)	0.022	0.08	(0.01–0.73)	0.025	0.33	(0.08–1.36)	0.125	0.54	(0.08–3.42)	0.509
Gender												
Male		(ref)			(ref)			(ref)			(ref)	
Female	2.21	(0.62–7.80)	0.219	1.46	(0.39–5.47)	0.577	0.56	(0.16–1.96)	0.367	0.41	(0.08–2.09)	0.280

CI: confidence interval; OR: odds ratio.

### Fear of digital technology

The types and features of different digital technologies within the waiting room came up repeatedly during the focus groups' discussions. One key theme was the importance of choice for these patients due to their variable knowledge and experience of using digital technologies, as well as the physical limitations of their condition.

Participants described different levels of familiarity with digital technologies. Some discussed using tablets and applications (apps), referring to themselves as ‘technologically minded’; other participants described feeling uneasy and being ‘forced into’ using technology against their wishes. Participants who were less comfortable with technology felt that they would be nervous about using a tablet or computer kiosk in the waiting room but would welcome a paper leaflet.

I would probably veer away from a tablet. And I have got a tablet at home that I use all the time. Because I think in some way I am not really supposed to pick up *that* tablet.

But where I had something that was hard copy I think I would be very likely to pick that up if it was, you know, looked accessible. You could pick that up and have a look.

As with the question of face-to-face recruitment, age seemed to be linked to less familiarity with, or confidence using, digital technology in a public space, despite some experience of using it elsewhere. Expressions of interest in paper leaflets to take away may represent resorting to methods which have less attendant stress owing to their familiarity. Whether this would actually result in people enrolling with the registry or not is unclear, but the correlation between age and interests in digital technology is borne out by a younger participant's contrasting opinion in the following extract:

‘Me personally, no [to picking up leaflets in the waiting room]. I'm technology orientated a bit so I have my iPhone and my iPad and that does interest me. I would use that personally'.

### Physical limitations

Physical limitations imposed by their condition were highlighted by all participants. For some, these presented challenges in relation to the types of digital technology that might be suitable within a waiting room. It was clear from the transcripts that having a computer screen in a kiosk within the waiting room would not be a favourable option for this patient group due to having to walk to the kiosk and then stand whilst using it.

I am not sure whether I would have the confidence to actually… Because you are crippling a bit because of the drive, the long drive. You are crippled across the car park. You are sat in the chair and you think, ‘Oh, okay'. And you know the next time you are going to get up is for your appointment time and always you are stiff to get up and going.

In all honesty, I think I would be very reluctant to get up and cripple my way in front of everybody to get to that kiosk.

These concerns reflect these particular patients’ mobility difficulties. They demonstrate how patients manage their activity by planning their movements, but are simultaneously aware of their disability in front of others. It is not clear whether the novel activity of using a kiosk or table-mounted tablet would actually contribute a significant additional burden in terms of movement and discomfort, or whether this could be incorporated into these patients’ waiting time productively with consideration of positioning and access to the screens. These considerations also need to take other physical difficulties into account as the following extracts demonstrate. One participant, who suffered with arthritis in her hands and wrists, felt that having to hold a tablet would be difficult due to the weight:

‘The tablets themselves are very heavy and… you know, for me to hold them is actually [difficult]… Because I have arthritis in my hands…’.

Two participants described difficulties they experience with reading due to ophthalmic problems related to their musculoskeletal condition (rheumatoid arthritis); they explained how technology can help them to overcome these issues when reading, as the following extract demonstrates.

If I am outside I can read a paper or like here I can read the paper. But sometimes I can't, so like in a waiting room where there is no windows or anything. But my Kindle's got lines so I can read from that. And I guess it would be the same with an iPad or something, i.e. depending on the light behind it I may or may not be able to read it.

These suggestions indicate that planning the type and location of digital technology in clinical waiting rooms must take account of the constraints on the population who are being targeted to use them. It also indicates that kiosks, in particular, would be difficult for this population. Careful thought needs to be given to the height, weight and position of any device in a waiting room and to explore how devices could help to address the physical disabilities that this group of patients experience.

### Place and time

Participants were also asked where they would prefer to make the decision about signing up to OxPaRRM; whether they would prefer to do this whilst they wait in the clinic or in their home. Results are presented in Table 4. Approximately 50% reported being happy and 16% reported being unhappy about making a decision about signing up to OxPaRRM while attending the clinic. In contrast, over 80% reported being happy and 6% reported being unhappy about having to make a decision about signing up while at home. Results from ordinal logistic regression models indicated there were no relationships between the preferred location of making a decision to sign up to OxPaRRM and participant age or gender.

**Table 4.** Patient views on location of the Oxford Patient Research Registry – Musculoskeletal (OxPaRRM) decision.

	In clinic		At home	
	*n*	%	*n*	%
Happy	32	51	54	81
Neutral	11	17	6	9
Unhappy	10	16	4	6
Unsure	10	16	3	4

### Distractions in the waiting room

Participants' experience of being in the waiting room was found to be a very important concern for many patients, due to the regularity with which they attend clinics for follow-up. Participants described how they often felt distracted while they were in the waiting room and this was perceived negatively and positively, depending on the type of distraction.

Participants described feelings of anxiety prior to their appointment and attributed it to several factors, including wondering which doctor they would see, trying to remember symptoms and details of treatments to discuss during their appointment, concerns about waiting times and transport-related concerns such as parking fines and traffic jams delaying journeys home. Several participants felt that they were unable to concentrate in the waiting room prior to their appointment, because of the anxiety they experienced in anticipating the consultation.

I am in a state of anxiety before. Because I am wondering, ‘Are they going to change my medications?’ Or I am going there thinking, ‘I need to have them changed’. So I am going there rehearsing what I am going to say. So I almost zone-out of the waiting area and I am rehearsing what I am going to say.

These distractions and their attendant feelings of anxiety meant that some participants thought they would not be able to focus on or retain information they were given in the waiting room. This would mean that they would not be in a position to make an informed decision about signing up to a research registry. Several participants suggested having information that could be accessed later at home.

If you had paper with information on it with Web addresses and possible contacts where you could find out more then that is useful for somebody to take home with them. They could then go on to their own computer when they are ready… if you have only got a tablet there, you are not always going to remember what you have seen on the tablet unless there is some way in which you could send information to your email address or something.

By contrast, one participant stated that he would prefer to make the decision while in the clinic, as he would be unlikely to access that information at a later date.

I'd prefer doing it at the time at the clinic. That would be ‘my [preference] -' if I got an email, I get so many emails and so many spam emails that I personally would miss it… Yes, I'd prefer to do it at the point of the appointment.

However, some patients welcomed distractions in the waiting room, perceiving them to be a positive way of avoiding stresses associated with waiting. In particular, reading was used as a coping mechanism by some. One participant described her approach to waiting.

‘I don't really think. I just take my Kindle and I just read. And I don't care how long I have got to sit there really. I just program myself to do that’.

Most participants expressed reluctance to use tablets or kiosks in a waiting room but suggested a preference for conventional information leaflets to follow up on later. Interestingly, the youngest participant within the focus group sessions expressed support for using either a tablet or his own smartphone to access registry information and recruitment application whilst waiting in the clinic.

## Discussion

This study aimed to explore patients' views on engaging with digital technology to sign up to a research registry, while in the clinic waiting room. There have been a number of studies exploring the clinic waiting room as a potential space to promote key public health messages,^20,21^ conduct health education classes^22,23^ and to run interventions to reduce anxiety.^24^ While studies have produced mixed findings, there remains a growing consensus that the waiting room is a neglected area within clinical settings.^25^

While participants were positive about future opportunities to take part in research and use digital technologies to do this, the choices regarding how, where and when this happened were clearly crucial factors for many patients. The findings suggested that a range of options were required to suit varying needs of patients. It was clear from survey findings and from the focus group discussions that many patients wanted to have a choice about deciding to sign up for research either whilst still in clinic or at a later date.

Having the choice to make a decision at a later date seemed to be related to the psychological impact that being in the waiting room has on the patients; many felt they were distracted due to anxiety relating to their upcoming appointment and therefore not be in a position to objectively consider information or give informed written consent. Previous evidence has indicated that patients in primary care settings may feel distress within the waiting room. A qualitative study conducted with 60 GPs across France reported that they expressed concern about how stressful the waiting room can be for patients.^8^ Similar findings have been reported in a study conducted in Italy which explored experiences of patients with cancer who were waiting for clinical appointments and treatments within hospital settings. More than 80% of patients felt there was an emotional cost to waiting, and almost one-third of all patients perceived this cost to be either high or very high. Subsequently, patients welcomed alternative activities to distract them whilst waiting; these included a television screen, library or discussions with healthcare professionals.^26^

A desire for distractions whilst in the waiting room is of particular interest in light of the previous recruitment rate to OxPaRRM; 97% of patients who were approached and invited face-to-face by a RN agreed to sign up to the registry. While we cannot rule out the possibility that patients may feel coerced, one plausible reason for this may be that patients are viewing the discussion about the registry as a welcome distraction; they may feel calmer and therefore, more able to make a decision about signing up while in the clinic waiting room. However, it is unclear how many people would sign up when they return home, or if they would be distracted by other things.

An additional reason for the high OxPaRRM recruitment rate may be due to the high level of trust that patients place in RNs.^27^ In an article that reflects on the lessons learnt from the New Jersey Family Medicine Research Network (NJFMRN) study, Felsen et al. describe the importance of building rapport with patients for recruitment into research studies, particularly those who were feeling unwell or in pain at the point of recruitment. Acknowledging patients' pain and discomfort, while explaining the purpose of the study, increased recruitment rates to the NJFMRN study.^28^ It is likely that RNs are more sympathetic and sensitive to patients' symptoms and this may have contributed to the high recruitment rate previously observed in the OxPaRRM study.

Providing an option on a digital tool to enable patients to sign up to OxPaRRM at a later date could be addressed fairly easily by including a function that forwarded information to interested patients who provide their contact details or by simply giving them a leaflet with the Web address. However, this does raise questions about whether patients would actually be motivated enough to access information later at home, unprompted, because evidence from web-based interventions indicates that attrition is high, particularly with harder to reach populations.^29,30^

As age increased, patients were more likely to prefer a face-to-face invitation to the research registry when in the waiting room and the need for information in both digital and traditional, paper-based formats was a clear and consistent theme throughout the focus group discussions. Many patients cited a fear of digital technology as a key reason for the addition of a hard copy option. A low proportion of participants in this study were less than 41 years old. Evidence suggests that as age increases, the likelihood of engaging with digital technology and using the Internet decreases. In addition to this, having a lower income, perceived poorer health and being female are also associated with less use in elderly populations.^31^ Other studies exploring the use of digital technology by older populations within healthcare have reported low uptake, with participants citing fear as one of the main deterrents, despite being offered emotional and technical support to do so.^32,33^

Despite this, a large proportion of participants were between 41 and 60 years old, and likely to regularly interact with digital technologies. Therefore, physical limitations imposed by their musculoskeletal conditions may account for their support of information available in both digital and hard copy format. Patients discussed the physical limitations imposed on them by their musculoskeletal conditions as a reason for not engaging with different types of digital technology in the waiting room. It was clear from the discussions that a kiosk or check-in type screen would not be suitable because patients felt they would have difficulty walking to and standing next to these types of devices. By contrast, some patients described how digital technologies were easier to use compared to hard copies because of the symptoms of their condition.

Consideration must be given to these issues before any digital technologies are introduced to waiting rooms to prevent alienating certain populations of patients; using only digital methods may have a detrimental impact on recruitment rates. However, these physical limitations are fairly specific to the sample population within this study, and it is possible that patients attending GP practices or family planning clinics, where the patient demographics are more varied, will have different perceptions and expectations of digital technology use within clinic waiting rooms.

Personal interactions, described as ‘the personal touch’, were an important feature for participants and it was apparent that they wanted to know what was happening, why it was happening and who would be doing it. In a study exploring participants’ perspectives of taking part in an Internet-based trial, the authors reported that one of the most frequently reported disadvantages to an Internet-based study was the inability to ask questions or seek clarification. Other frequently reported disadvantages to this format were the lack of personal contact and participation feeling less rewarding compared to more traditional models of research.^34^

The importance of being informed about the study outcome was clear from the focus group discussions and this appealed to participants' sense of being valued. This has been reported in a number of studies; a systematic review of studies exploring the types of information that participants wanted to know about research found that 91% of participants wanted to know the outcome of the study.^35^ However, a study exploring health research participants' expectations on this reported inconsistencies between people's preferences and researchers' willingness to share findings.^36^ When this feature has been tested in trials, the outcome was not as positive as originally expected. A study with cancer trial patients reported that many participants were confused regarding the results and disappointed that the aggregated findings were provided, rather than personal results.^37^

Some studies have reported that feeding back study findings can even be harmful to some patients,^37,38^ therefore this must be done with great care; potential participants and researchers should work together collaboratively to make these decisions before the study begins.^36^ The majority of people who participate in research studies do so for altruistic reasons. Therefore, if people do not feel their time and effort are valued, there is a risk they may not participate in future studies.

An unexpected finding in this study was that participants discussed the research registry as a way of starting conversations with other patients in the waiting room. Opportunities to meet other patients with similar conditions and connect with peers appeared to be important. Identity is socially prescribed and constructed through an individual's experiences, beliefs and values. Experiencing a chronic illness can change one's perceptions of oneself and a new reality is constructed.^39^ Therefore, social networks can play a crucial role in how patients perceive their disease state as knowledge passed on from peers is experiential rather than from formal training.^40^ This could be used to rethink the design of waiting rooms and how they might be used to support interactions between patients with similar conditions. In addition to this, there may be potential for peer-to-peer recruitment strategies to be explored within research studies if clinicians are not available to perform this task. However, this would require sufficient training and many subject areas may not be suitable due to the complexity or the sensitive nature of the study.

The importance of peer support in the treatment of chronic conditions is well recognised by researchers and healthcare professionals, and there are many peer support interventions that have been developed to take advantage of that relationship to deliver healthcare education or emotional support.^41^ The evidence from our study suggests that the design of the electronic interface for recruiting patients to a research registry of this kind should capitalise on the social aspects of being part of a group with similar conditions. Potential ways to do this might include a link to details of peer support group meetings and online discussion groups. For those who do not feel comfortable using digital technology, simply including a list of recognised support groups on the back of a research registry leaflet may provide useful.

The main strength of this study is the mixed methods design; the focus group discussions provided explanations for some of the key survey findings. In addition to this, by designing a short questionnaire we were able to recruit participants whilst they were waiting for their appointments and increase the sample size. This also meant participants were answering questions relating to the clinic waiting room whilst they were in it, potentially providing more accurate responses. However, we acknowledge that based on the findings of this study, responses may be less accurate due to distractions and anxiety caused by the experience of waiting or due to patients experiencing pain as a result of their condition. Unfortunately, we were unable to collect data on pain and fear of pain experienced within the waiting room due to time limitations, but acknowledge that this would be interesting to explore as an effect modifier. Additional limitations of this study include the small sample sizes recruited in each phase of the study which mean that the findings are unlikely to be generalisable to all patients with musculoskeletal conditions in England. However, the findings do indicate that there are populations of patients who would be interested in engaging with digital technologies within the waiting room. Finally, we acknowledge that we only recruited musculoskeletal patients, who are more likely to be older and experience a range of symptoms, meaning the viewpoints of these patients may differ greatly from younger patients in different healthcare settings, such as primary care.

## Conclusions

Overall, the findings from this study have indicated that many patients with musculoskeletal conditions in the UK may be interested in learning about opportunities to participate in research whilst using digital technologies within the waiting room. The results also highlighted the need for choice regarding the presentation and format of information, the devices used and whether it can be access at a later date whilst at home. However, before methods such as this are implemented in clinic waiting rooms, we recommend that further research is conducted to explore whether patients' perspectives differ within more varied clinical settings or with a varied demographic of patients, and whether patients would actually access a website at a later date in their homes, were the option available.
